# Treatment of Prolapsus Ani in Children

**Published:** 1876-09

**Authors:** 


Treatment of Prolapsus Ani in Children.—Prof. Henoch, of Berlin, treats this affection very successfully with hypodermic injections of strychnia and ergotine. He dissolves $\frac{7}{10}$ of a grain of sulphate of strychnia in $2\frac{1}{2}$ drachms of distilled water, and injects daily, in the neighborhood of the anus, from three to six minims—$\frac{1}{70}$ to $\frac{1}{35}$ grain strychnia. This remedy is in some cases remarkably successful, but in other cases it fails entirely. Ergotine seems to act more certainly. He injects daily a quarter, or a half, or even an entire syringeful (Pravaz's) of a solution of the strength of one to ten. The professor advises that when the child goes to stool, the chamber be arranged on a table or chair, so that the legs will hang free. This will prevent, to a great extent, the straining action of the abdominal muscles.—*Allgemeine Medicinische Central Zeitung*, May 31st, 1876.

